# Supplementary material for: Bayesian refinement of protein structures and ensembles against SAXS data using molecular dynamics
Source: PLoS Comput Biol. 2017 Oct 18;13(10):e1005800. doi: 10.1371/journal.pcbi.1005800 (PMC5662244; doi:10.1371/journal.pcbi.1005800)
Supplement: S1 Archive — (BZ2) [file pcbi.1005800.s011.tar.bz2 › gromacs_4.62_bayesian_saxs_refi/share/html/online/g87.html]

Gromos87 trajectory file format


## g87 file format

|  |  |
| --- | --- |
| Main Table of Contents | **VERSION 4.6** |
| GROMACS homepage | **Sat 19 Jan 2013** |

---

This is a simple ASCII format:  

```
TITLE
x(i) (10f8.3)
box(m) (10f8.8)
```

The number of atoms is **not** stored, neither is there any indication
of whether a box is present. The box is stored only for constant pressure
simulations. So, you simply have to remember this stuff.

---

http://www.gromacs.org
